# Identification and Synthesis of Putative Pheromone Components of the Threatened Salt Marsh Bagworm Moth, *Whittleia retiella* (Lepidoptera: Psychidae)

**DOI:** 10.1007/s10886-020-01145-x

**Published:** 2020-02-13

**Authors:** Rizan Rahmani, David Carrasco, Glenn P. Svensson, Hartmut Roweck, Nils Ryrholm, Mattias C. Larsson, Erik Hedenström

**Affiliations:** 1 Eco-Chemistry, Department of Chemical Engineering, Mid Sweden University, SE-851 70 Sundsvall, Sweden; 2 Department of Plant Protection Biology, Swedish University of Agricultural Sciences, Box 102, SE-230 53 Alnarp, Sweden; 3 Present Address: MIVEGEC, University of Montpellier, IRD, CNRS, Montpellier, France; 4 Department of Biology, Lund University, Lund, SE-223 62 Sweden; 5 Faculty of Agricultural and Nutritional Sciences, Christian Albrecht University, Olshausenstraße 75, R. 220 Kiel, Germany; 6 Department of Electronics, Mathematics and Natural Sciences, Faculty of Engineering and Sustainable Development, University of Gävle, SE-801 76 Gävle, Sweden

**Keywords:** Endangered species, Species monitoring, Field observation, 1-Methylethyl (5Z)-dec-5-enoate, (1*S*)-1-Methylpropyl (5*Z*)-dec-5-enoate, Stereoisomers, Enantiomers

## Abstract

*Whittleia retiella* (Newman, 1847) is a threatened salt marsh species of the bagworm moth family Psychidae. For its preservation it is necessary to develop efficient tools to survey its distribution and habitat requirements in order to use appropriate conservation methods. Such tools may be pheromone-based monitoring systems, which have documented efficacy in establishing the occurrence of cryptic insect species in nature. By using gas chromatography combined with electroantennographic detection (GC-EAD), we found two compounds in female *W. retiella* headspace samples and whole-body extracts that elicited electrophysiological activity in male antennae. Gas chromatograpy coupled with mass spectrometry (GC-MS) operating in electron impact (EI) mode and comparison of the analytical data with those of synthetic reference compounds showed the chemical structures of these putative pheromone components to be (1*S*)-1-methylpropyl (5*Z*)-dec-5-enoate and 1-methylethyl (5*Z*)-dec-5-enoate. Field assays using baits loaded with synthetic compounds revealed that conspecific males were attracted to (1*S*)-1-methylpropyl (5*Z*)-dec-5-enoate alone or in combination with 1-methylethyl (5Z)-dec-5-enoate, whereas 1-methylethyl (5Z)-dec-5-enoate neither attracted nor repelled males in the field assays when tested alone. This study shows the potential of using (1*S*)-1-methylpropyl (5*Z*)-dec-5-enoate for monitoring *W. retiella* to gather more detailed information about the geographic distribution and habitat needs of this rare moth.

**Electronic supplementary material:**

The online version of this article (10.1007/s10886-020-01145-x) contains supplementary material, which is available to authorized users.

## Introduction

Insect sex pheromones have traditionally been studied for the purpose of pest control (Witzgall et al. 2010). More recently, significant efforts have been made to develop pheromone-based monitoring systems also for rare and threatened species as a novel tool in conservation biology (Larsson 2016). Sex pheromones can be used to detect target species even at very low population densities and thus have high potential for monitoring population dynamics, habitat needs, and changes in the geographic distribution of red-listed insect species, which would otherwise be difficult to assess. Access to efficient monitoring systems may, in turn, allow to serve as indicators of the conservation status of habitats for these species that are in danger of being destroyed or degraded.

The majority of sex pheromone identifications in rare and threatened insects have so far been limited to a few groups, predominantly beetles. These include scarabaeids such as the hermit beetle species *Osmoderma eremita* and *Osmoderma barnabita* (Larsson et al. 2003; Svensson et al. 2009), which constituted the first model species for pheromone identifications specifically for conservation purposes, and the noble chafer *Gnorimus nobilis* (Harvey et al. 2018), several click beetle species such as *Elater ferrgineus* (Tolasch et al. 2007), *Betarmon bisbimaculatus* (Koenig et al. 2015), and *Idolus picipennis* (Koenig et al. 2016) and longhorn beetles such as *Prionus coriarius* (Barbour et al. 2011), *Rosalia alpina* (Kosi et al. 2017), *Phymatodes pusillus* (Molander and Larsson 2018), and *Plagionotus detritus* (Molander et al. 2019). To date, only a limited number of rare and endangered moth species have been investigated *e.g.* the Spanish moon moth *Graellsia isabellae* (Millar et al. 2010), and the clearwing moth *Synanthedon vespiformis* (Burman et al. 2016).

The bagworm family Psychidae includes approximately 1000 species of small to medium sized moths. A distinct characteristic of this archaic family is that individuals complete larval development within a self-enclosing bag (Heppner 2008), and females of many species are wingless or have reduced wings. Unlike most moth species, which emit the sex pheromone from a gland at the tip of the abdomen, female bagworms release their pheromone from specific gland cells located on the thorax and/or the anterior part of the abdomen (Leonhardt et al. 1983; Subchev et al. 2000). The Psychidae include several important pests, but also rare and endangered species. One such species, whose conservation may benefit from pheromone monitoring and which would potentially constitute a good indicator species for landscape preservation is *Whittleia retiella* Newman, 1847 (Lepidoptera: Psychidae). Adult males of *W. retiella* have a wingspan of 7.5–8.5 mm, whereas females are wingless with rudimentary pairs of legs (Rickert et al. 2009). The species is a salt marsh specialist, whose larvae use grasses such as *Puccinellia maritima* (Poaceae) to feed on and eventually pupate. This rare halobiotic moth flies at the end of April or the beginning of May (Bengtsson and Palmqvist 2008; Ryrholm 2005). It is listed in the Red List of endangered animals of Germany as very rare (Binot et al. 1998) and in Sweden as critically endangered (Ahrné et al. 2015).

*W. retiella* could be considered an important indicator species for salt marshes that extend along the North Sea coast from Denmark to the Netherlands. Salt marshes are characterised by shifting tides and varying salt concentrations, providing a habitat for a number of characteristic species as well as constituting breeding grounds and a stopover habitat for migrating waders and shorebirds. The salt marshes are products of long periods of management by grazing, which is prerequisite for their long-term persistence. An important aspect of their preservation is to manage grazing intensity to maintain high biodiversity. Because *W. retiella* appears to be largely dependent on relatively well-managed marshland for its persistence, it could serve as an indicator for favourable management (Rickert et al. 2009; Rickert 2010). Due to its minute size and clandestine lifestyle, the species is extremely hard to find, and a pheromone would thus be a valuable tool for monitoring of *W. retiella* and the quality of its habitats.

In the present study, we identify two compounds, (1*S*)-1-methylpropyl (5*Z*)-decenoate and 1-methylethyl (5Z)-decenoate, released by female *W. retiella*, which are sex pheromone component candidates based on the electrophysiological and behavioural activity they trigger in conspecific males. Syntheses and analytical data of these compounds and compounds used for reference are also presented in this work.

## Methods and Materials

### Collection of Moths

Specimens were collected in 2010 and 2012 from the salt marshes and grasslands by the North Sea coast at Westerhever, Nordfriesland, Germany. Larval and pupal cases made of small fragments of dry grass spun together were collected, and larvae were sorted by weight and separated by sex. Generally, females were heavier than males. Larvae were kept in Petri dishes together with living plants of *P. maritima* to feed on until pupation. Emerging males were collected and placed in plastic tubes with cotton stoppers in the fridge until use for electrophysiological analyses. Female pupae were either left inside their pupal cases and used for repeated odour collections or carefully removed from their cases using thin forceps for direct extraction (see below).

### Collection of Putative Pheromone Components

Between 6 and 42 adult females (less than one week old) were pooled into 20 mL glass scintillation vials with two holes made in the lids. One hole was fitted with an activated charcoal filter through which air entered the vial, while the other was fitted with an odour collection filter made from Teflon tubing (60 mm with I.D. 3.0 mm O.D. 4.0 mm) and containing 25 mg Porapak Q (PQ) mesh size 80/100 (Sigma-Aldrich, St. Louis, MO, USA) as adsorbent material. Air was pumped through the vial and the collection filter at 0.2 L/min^-1^ for several hours (range: 17–24 hr) during 4 consecutive days, using a KNF NMP830KNDC air pump (KNF Neuberger, Balterswil, Switzerland). Long and sequential collection times were justified since the calling time of the females during the day was unknown. The PQ filters were changed every day. After collection, the filters were extracted with 2 × 150 μl redistilled *n*-hexane, which was collected in 1.5 mL glass vials and stored at −20 °C until further use. In addition, 15 females were removed from their old pupal cases and extracted for 30 min in 1 mL *n*-hexane, which was later concentrated to 300 μl under N_2_ and stored in 1.5 mL glass vials at −20 °C until further use.

### Electrophysiology

In order to screen for putative pheromone components, headspace samples and whole-body extracts of female moths (less than one week old) were analysed using combined gas chromatography and electroantennographic detection (GC-EAD) (Larsson and Svensson 2005). The head of a male moth with both antennae was mounted to a PRG-2 EAG Probe (10 × gain) (Syntech, Kirchzarten, Germany) using conductive gel (Blågel, Cefar, Malmö, Sweden). The GC effluent passed through a heated transfer line set at 255 °C and was mixed with charcoal-filtered and humidified air before reaching the antennal preparation, which was positioned 1 cm from the glass tube outlet. Extracts or headspace samples (2 μl) were injected into an Agilent 7890A gas chromatograph (Agilent Technologies, Palo Alto, CA, USA), equipped with a polar HP-INNOWax column (30 m × 0.25 mm I.D. × 0.25 μm film thickness; J&W Scientific, Agilent Technologies, Santa Clara, CA, USA) operated in splitless mode. Hydrogen was used as carrier gas at a flow rate of 1 mL·min^−1^, and the injector temperature was 250 °C. The GC effluent was split at a 1:1 ratio between the flame ionisation detector (FID) and the antennal preparation. The oven temperature was maintained at 50 °C for 1 min after injection and then increased to 210 °C at a rate of 10 °C·min^−1^ and a final hold of 10 min. Additional analyses were performed on an HP 6890 N gas chromatograph equipped with a non-polar HP-5 column (30 m × 0.32 mm I.D. × 0.25 μm film thickness, J&W Scientific). For these analyses, an antenna was mounted between two glass capillaries filled with Beadle-Ephrussi ringer solution and connected to a pre-amplifier (10 × gain) (Syntech). The GC effluent passed through a Gerstel ODP-3 transfer line, which was temperature adjusted in tandem with the GC oven temperature. It was mixed with charcoal-filtered and humidified air before reaching the antenna, placed 0.5 cm apart from the glass tube outlet. Hydrogen was used as carrier gas at an average linear flow of 45 cm·s^−1^ and an injector temperature of 225 °C. The GC effluent was split 1:1 in a Gerstel 3D/2 low dead volume fourway-cross (Gerstel, Mülheim, Germany) with 4 psi of nitrogen added through the fourth connection. The oven temperature was maintained at 50 °C for 3 min after injection and increased by 10 °C·min^−1^ to 230 °C, with a final hold of 10 min. All recordings were performed using the GC-EAD Pro ver. 4.1 software (Syntech).

### Chemical Analysis

Tentative identification of the female-produced, antennally active compounds was performed by GC-MS with column types matching the columns used for GC-EAD recordings. An HP-5 ms capillary column (60 m × 0.25 I.D. × 0.25 μm film thickness, Agilent Technologies) was mounted in a GC 6890 N model GC interfaced to a 5975 mass selective detector (Agilent Technologies, Palo Alto, CA, USA). The carrier gas was helium with a constant flow rate of 1.8 mL·min^−1^ (inlet pressure 172 kPa) at an injector temperature of 225 °C. Analyses started at 30 °C with a 3 min hold and programmed to 250 °C at a rate of 8 °C·min^−1^. The transfer line temperature started at 150 °C and was then set to remain 10 °C above the oven temperature. Sample injections of 2 μL of extracts were made manually in splitless mode (split vent opened after 0.5 min). Headspace samples were also analysed using an HP 5890II gas chromatograph linked to an HP 5972 mass spectrometer equipped with an HP-INNOWax column (30 m × 0.25 mm I.D. and 0.25 μm film thickness; J&W Scientific). Helium was used as the carrier gas at a velocity of 40 cm·s^−1^ and an injector temperature of 220 °C. After injection in splitless mode, the oven temperature was maintained at 50 °C for 2 min and then increased by 10 °C·min^−1^ to 250 °C, with a final hold for 10 min.

More detailed investigations of insect extracts were performed on a Hewlett-Packard 6890 N GC using a HP 5973 mass spectrometer operating in electron impact mode (EI, 70 eV). Helium was used as the carrier gas (flow rate = 1.0 mL·min^−1^), and the split/splitless injector was operated in splitless mode for 2.5 min at 250 °C. The transfer line was maintained at 250 °C, and the MS source was set to 230 °C. Two different capillary columns were used: i) a 30 m × 0.25 mm I.D. × 0.25 μm film thickness fused silica capillary column VF-23 ms (Agilent Technologies); temperature program from 50 °C to 230 °C at a rate of 10 °C·min^−1^, then held at 230 °C for 10 min and, ii) a 30 m × 0.25 I.D. × 0.25 μm film thickness fused silica capillary column CP-WAX 58 (Agilent Technologies); initial temperature 50 °C, held for 2 min, then increased to 100 °C at a rate of 10 °C·min^−1^, and then held at 100 °C for 35 min.

For the separation of enantiomers, a Hewlett-Packard 6890 N GC linked to a HP 5973 mass spectrometer was used with helium as the carrier gas (flow rate = 1.0 mL·min^−1^). The split/splitless injector was operated in splitless mode and set at 250 °C. The transfer line was maintained at 250 °C, and the MS source was set to 230 °C. The GC was equipped with a fused silica capillary column (30 m × 0.25 mm ID × 0.25 μm film thickness) coated with Cyclosil-B (Agilent Technologies). Separations were performed using a temperature program starting at 50 °C (2 min hold), then increased to 250 °C at a rate of 5 °C·min^−1^.

### Data Analysis

The raw MS data were analysed using the Workstation v7.0.0 program (Agilent). Compounds were analysed by comparing the mass spectra of natural products with data reported in the NIST MS 2.0 mass spectral library, using reverse and forward match values. The postulated structures were also confirmed by comparing retention times and mass spectra of the natural compounds with those of synthetic samples.

### Commercial Chemicals

(4*E*)-Dec-4-enoic acid was obtained from Penta International Corporation company, (4*Z*)-dec-4-en-1-ol and (5*E*)-dec-5-en-1-ol were purchased from TCI Europe, and (5*Z*)-dec-5-en-1-ol was obtained from Bedoukian Research Inc., Danbury, CT, USA. *Candida rugosa* lipase (CRL), (5-carboxypentyl)triphenylphosphonium bromide, and racemic butan-2-ol and propan-2-ol were purchased from Sigma-Aldrich, whereas (2R)-butan-2-ol and (2S)-butan-2-ol were obtained from Alfa-Aesar (Haverhill, Massachusetts, USA). All chemicals and solvents were of the highest available purity *i.e.* >95%.

### Synthesis of Acids

(4*Z*)-Dec-4-enoic acid, (5*E*)-dec-5-enoic acid, and (5*Z*)-dec-5-enoic acid were prepared via oxidation of (4*Z*)-dec-4-en-1-ol, (5*E*)-dec-5-en-1-ol, and (5*Z*)-dec-5-en-1-ol, respectively, according to Bowden (Bowden et al. 1946). Dec-6-enoic acid was synthesised via Wittig reaction from (5-carboxypentyl)triphenylphosphonium bromide, sodium hexamethyldisilylamide (NaHDMS), and butanal in THF according to Wube (Wube et al. 2011). For details of the syntheses and analytical data see Supplementary Material.

### General Synthesis of Reference Esters

In typical Fischer-Speier esterification reactions a carboxylic acid, an alcohol (in excess amount), and a catalytic amount of sulfuric acid (2 M) were mixed in a round-bottom flask. The contents were heated on a boiling water-bath and stirred for different lengths of time. For details of the syntheses and analytical data see Supplementary Material.

### Lipase Catalyzed Esterification

A facile enzymatic esterification process using crude lipase CRL as a biocatalyst in an organic solvent for the direct synthesis of (2*S*)-1-methylpropyl (5*Z*)-dec-5-enoate and (2*R*)-1-methylpropyl (5*Z*)-dec-5-enoate. (5*Z*)-Dec-5-enoic acid and (2*R*)-butan-2-ol as well as (2*S*)-butan-2-ol were used according to Sabbani et al. (2007) and Chang and Hsu (2003). For description of analytical data and the synthesis see Supplementary Material.

### Preparation of Baits

Lures with synthetic pheromone candidate compounds were prepared by dissolving known amounts of compounds by weight in redistilled *n*-hexane. Samples were prepared to obtain single-compound solutions or blends with desired amounts of test compounds. Solutions were pipetted as 100 μl aliquots into the cups of hollow red rubber septa (11 × 5 mm, #224100–020; Wheaton Science Products, Millville, NJ, USA) and the hexane allowed to evaporate in a fume hood after being partially adsorbed into the septum. Two sets of baits were prepared (Table 1). The first set was originally prepared in 2015 and included a broad range of combinations of the racemate and enantiomers of 1-methylpropyl (5*Z*)-dec-5-enoate. The second set, prepared in 2016, included a more limited number of blends comprising only the *(S)-*enantiomer. Each bait type was individually marked with a felt-tip pen. Baits were stored individually in the freezer when not in use and were brought out in the field for short periods (a few hours per day) during approximately one week during each season over several years.

**Table 1:** Two different series of lures were prepared in 2015 (bold) and 2016 (italic), respectively

Series	Compound	Blank	1	2	3	4	5	6	7	8	9	10	11	12
Series 1	Isoprop-C5DA			10	30		30	10	30	100		10	30	50
2015-	(rac)-2b-C5DA					200	200	100	100					
	*(S)*-2b-C5DA										100	100	100	100
	*(R)*-2b-C5DA		100	100	100									
	Blank	Hexane												
Series 2	Isoprop-C5DA									100		10	30	50
2016-	*(S)*-2b-C5DA										100	100	100	100
	Blank	Hexane												

Both sets of lures were used to varying extent from the years they were prepared. Numbers represent μg loading of different synthetic compounds onto the septa. Blank baits were loaded with solvent (*n*-hexane) only. Isoprop-C5DA = 1-methylethyl (5*Z*)-dec-5-enoate, 2b-C5DA = 1-methylpropyl (5*Z*)-dec-5-enoate. Different enantiomeric composition of 2b-C5DA (*S*, *R* or racemic) is indicated

### Field Bioassays

Attempts to observe the attraction of male *W. retiella* to synthetic baits were performed in 2015–2018 at several localities in Denmark in biotopes corresponding to preferred habitats of the species (NW Jutland: Hjelm Hede in Skive. NE Jutland: Mulbjerge at Dokkedal. Central Jutland: Kongenshus Hede and Gindeskov Krat, near Viborg. Dybdal Bæk, near Nørre Snede. E Jutland: Sejs Hede near Silkeborg). Observations of flying *W. retiella* at these localities are rare, and populations were expected to be very small. Therefore, no attempts were made to trap any insects with baits, but were based on observations of free-flying males approaching the different lures. Attempts to observe flights and attraction of *W. retiella* were limited to days of suitable weather with sun and limited wind during the expected short flight season in May–June. Behavioural observations of male attraction were performed by placing a series of rubber septa on the ground with individual septa at a distance of 50–100 cm apart. Each septum was marked with an individual number or letter with a felt-tip pen, but the content of each septum was unknown to the observer. At each visit, the septa were displayed from one to several hours. Flying males approaching the septa were counted on each occasion, and their flight patterns with repeated approaches to individual septa were noted.

## Results

### Electrophysiology

**Fig. 1:**
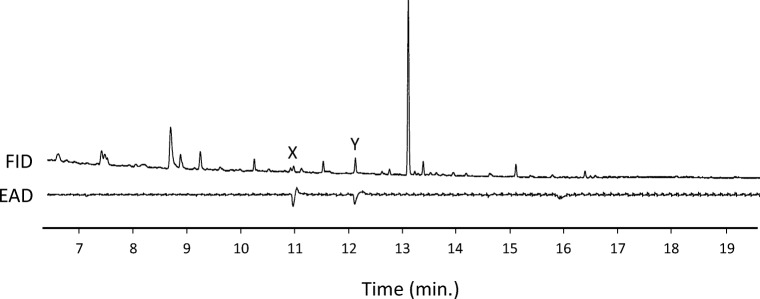
Coupled gas chromatographic-electroantennographic (GC-EAD) analysis of a headspace sample from females of *Whittleia retiella*. The upper trace shows the response of the flame ionization detector (FID), and the lower trace shows the antennal response (EAD) of a conspecific male. The analysis was perfomed using a polar HP-INNOWax column revealing the presence of two unknown physiologically active compounds, X and Y

At first, we investigated the electrophysiological activity of male antennae of *W. retiella* when stimulated with female-produced compounds collected by headspace sampling or whole-body extraction*.* The GC-EAD analysis on a polar column (HP-INNOWax) showed that two compounds (X and Y), sampled with both methods, elicited electrophysiological activity in male antennae, indicating that they are candidates as sex pheromone components of *W. retiella* (Fig. 1). To minimise the possibility of overlapping peaks, the GC-EAD analysis was repeated on a less polar GC-column (HP-5), which again resulted in antennal responses to two female specific compounds (data not shown).

### Chemical Analysis

**Fig. 2:**
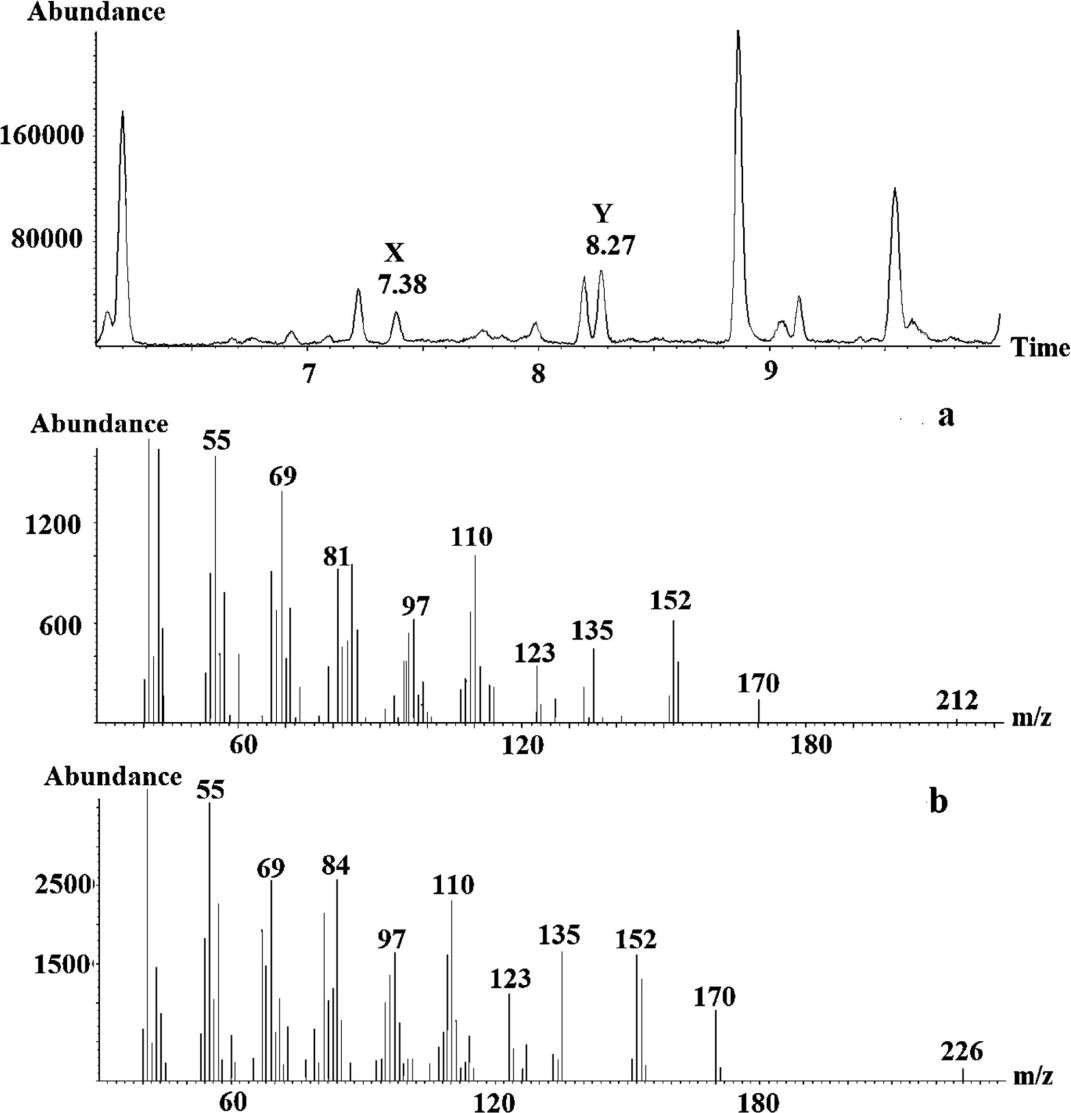
Part of the gas chromatogram (Vf-23 ms column) of a *Whittleia retiella* female extract and the mass spectra of (**a**), the first unknown antennally active compound (X) eluting at 7.38 min and (**b**), the second unknown antennally active compound (Y) eluting at 8.27 min

Matching of the GC-EAD-active compounds with mass spectra were performed through comparisons of the peak profiles of GC-EAD-FID and GC-MS on the two different columns. Retention times of the earlier eluting minor component X and the later eluting major component Y differed significantly between the two columns, but the mass spectra of the two compounds matched on both DB-Wax and HP-5 columns. Subsequent GC-MS analysis of extracts was performed on a polar GC-column (Vf-23 ms). As seen in Fig. 2, the analysis showed highest masses at m/z 212 for compound X, eluting at 7.38 min and at m/z 226 for compound Y, eluting at 8.27 min, most probably representing the molecular ions (M^+^) of the two compounds. The observed losses of 42 amu from M^+^ = 212 and 56 amu from M^+^ = 226 produced the same fragment of m/z 170 for both X and Y (Fig. 2).

**Fig. 3:**
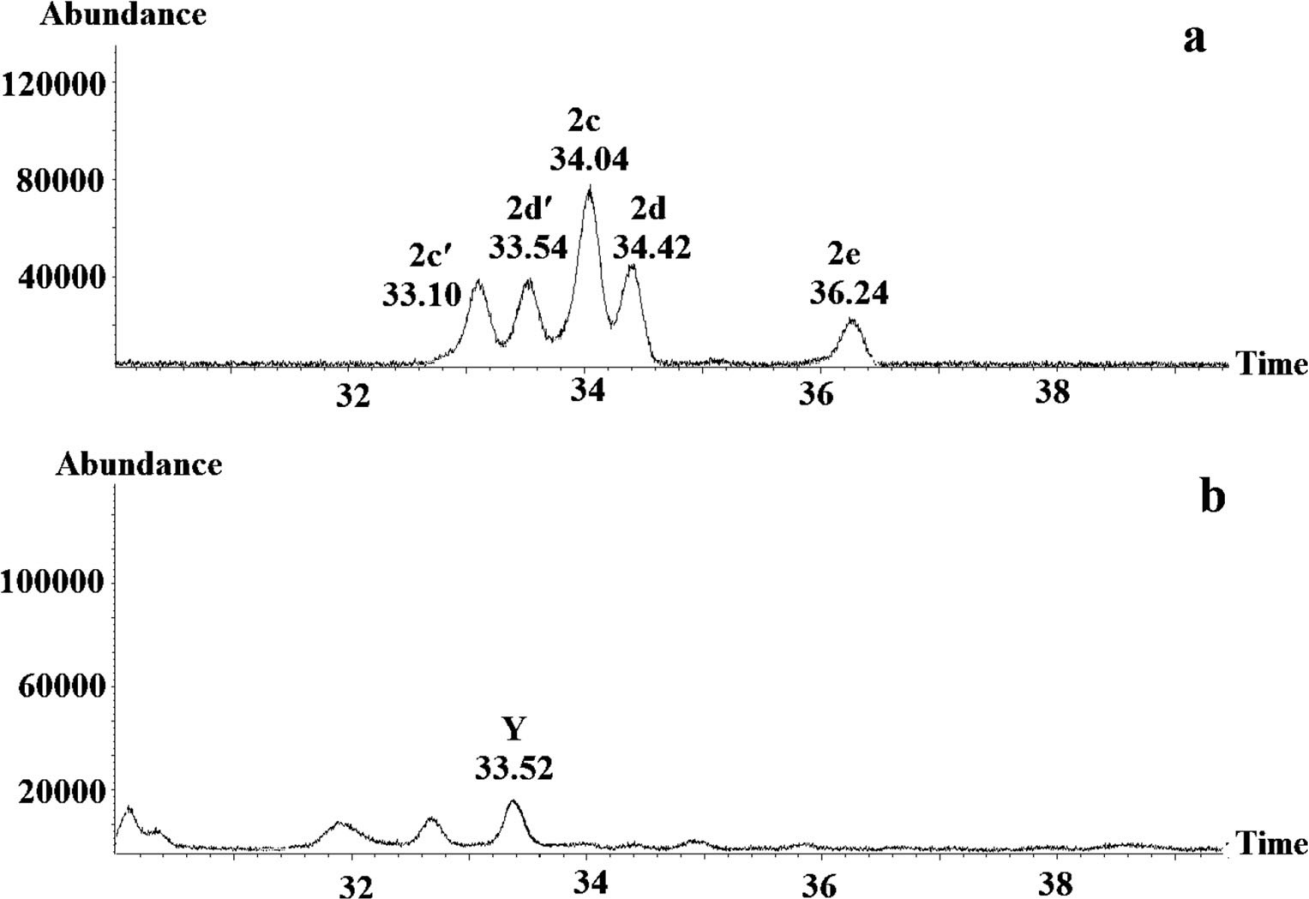
Part of the gas chromatogram (CP-WAX 58 column) of (**a**) 1-methylpropyl (4*E*)-dec-4-enoate (2c), 1-methylpropyl (4*Z*)-dec-4-enoate (2c’), 1-methylpropyl (5*E*)-dec-5-enoate (2d), 1-methylpropyl (5*Z*)-dec-5-enoate (2d’), and 1-methylpropyl *E*/*Z*-dec-6-enoate (2e) (**b**) *Whittleia retiella* female extract with compound Y eluting at 33.52 min

The daughter ion at m/z 170 showed the same fragmentation pattern for both compounds. In fact, it suggested a decenoic acid fragment, produced upon McLafferty rearrangement of carboxylic acid esters, which is supported by the presence of signals at m/z 153 (acylium ion) and m/z 152 (ketene fragment) (Francke et al. 2000). Consequently, the alcohol side of the esters X (MW 212) and Y (MW 226) would comprise 3 or 4 carbons, respectively. Checking the literature, revealed two plotted mass spectra of pheromones of Zygaenid moths: 1-methylpropyl (7*Z*)-tetradecenoate (Subchev et al. 1998) and 1-methylpropyl (7*Z*)-dodecenoate (Subchev et al. 2009). These spectra revealed fragmentation patterns similar to that of X and Y, which further supported our hypotheses. By analogy, and considering the structure of 1-methylethyl octanoate as another bagworm pheromone (Subchev et al. 2000), we concluded that the alcohol sides of X and Y could be represented by secondary alcohols rather than by primary ones. As a result, X was supposed to be a 1-methylethyl decenoate, whereas Y should be a 1-methylpropyl decenoate. However, the position of the double bonds remained to be determined. Very unfortunately, the small amounts of available natural material prohibited derivatisation, and we had to speculate: Considering that the biosynthetic pathways leading to the Zygaenid pheromones and to X and Y follow the same basic principles, it was conceivable to postulate that β-oxidation could chain shorten a (7*Z*)-C12-precursor to (5*Z*)-decenoic acid. Consequently, we synthesised 1-methylpropyl (5*Z*)-dec-5-enoate, and found that its retention time matched that of Y. Nevertheless, to be on the safe side, we synthesized the (5E)-isomer as well as *E*/*Z*-isomers of esters carrying the double bond in positions 4 or 6 and compared the retention times of these compounds with that of Y (Fig. 3).

**Fig. 4:**
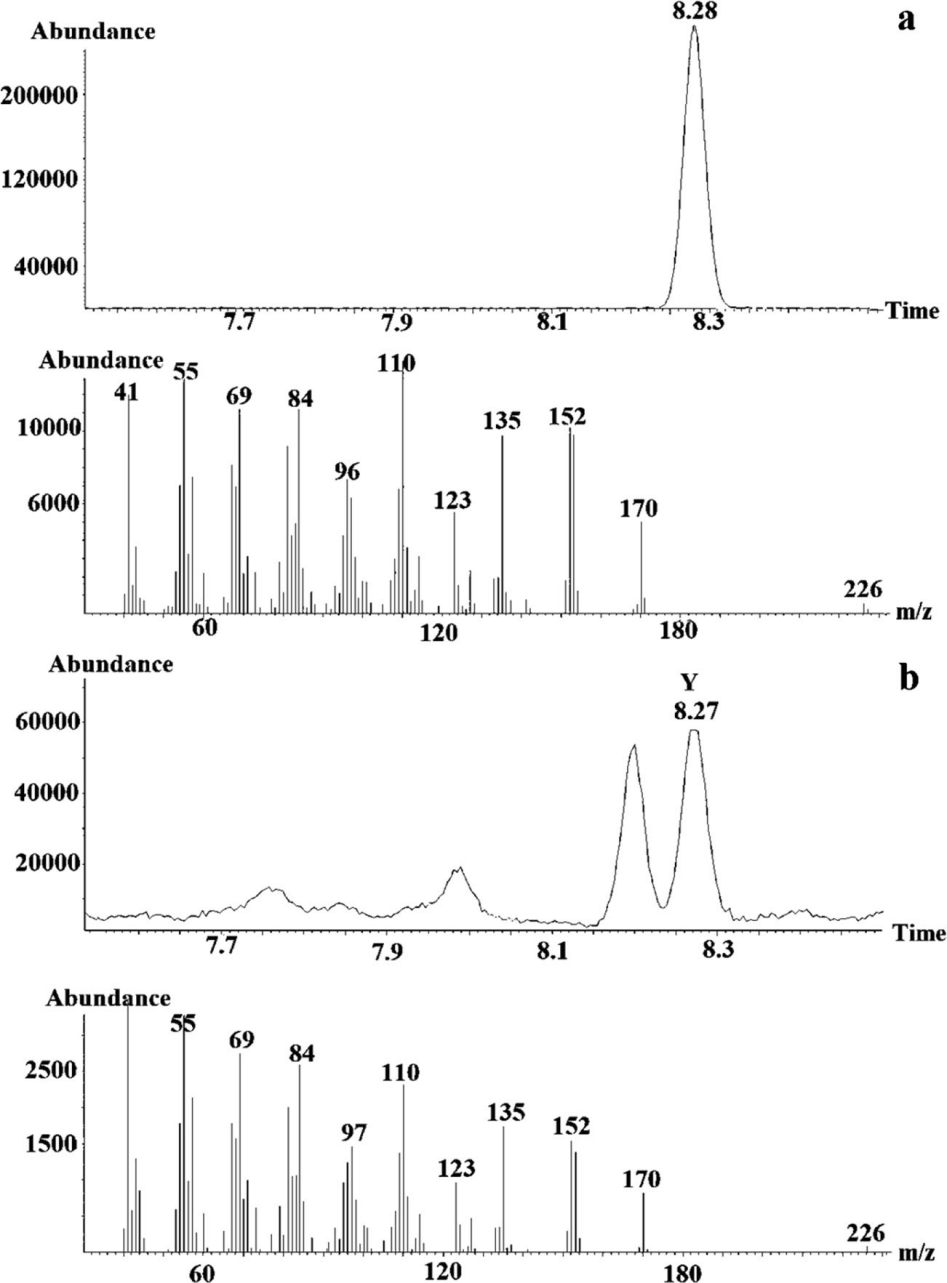
Part of the gas chromatogram (Vf-23 ms column) and mass spectra of (**a**) 1-methylpropyl (5Z)-dec-5-enoate and (**b**) compound Y, eluting at 8.27 min in the *Whittleia retiella* female extract

The retention time of 1-methylpropyl (5*Z*)-dec-5-enoate (2d’) coincided with that of compound Y in the female extract, whereas the other candidate compounds, including 1-methylpropyl (5*E*)-dec-5-enoate (2d), did not and were excluded from the investigation. As the mass spectra of 1-methylpropyl (5*Z*)-dec-5-enoate and that of the natural product Y were very similar, it was concluded that 1-methylpropyl (5*Z*)-dec-5-enoate represented compound Y, eluting at 8.27 min (Fig. 4).

**Fig. 5:**
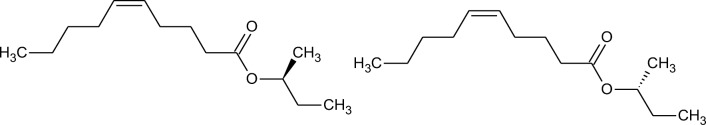
Chemical structures of the two enantiomers of 1-methylpropyl (5*Z*)-dec-5-enoate obtained via CRL catalysed esterification of (5*Z*)-dec-5-enoic acid using pure enantiomers of (2*S*)-butan-2-ol and (2*R*)-butan-2-ol, respectively

1-Methylpropyl (5*Z*)-dec-5-enoate has a chiral center and thus exists as two enantiomers (Fig. 5). To conclusively determine the absolute configuration of the *W. retiella* compound, both enantiomers were synthesised (see Supplementary Material for details) and submitted to enantioselective gas chromatography.

**Fig. 6:**
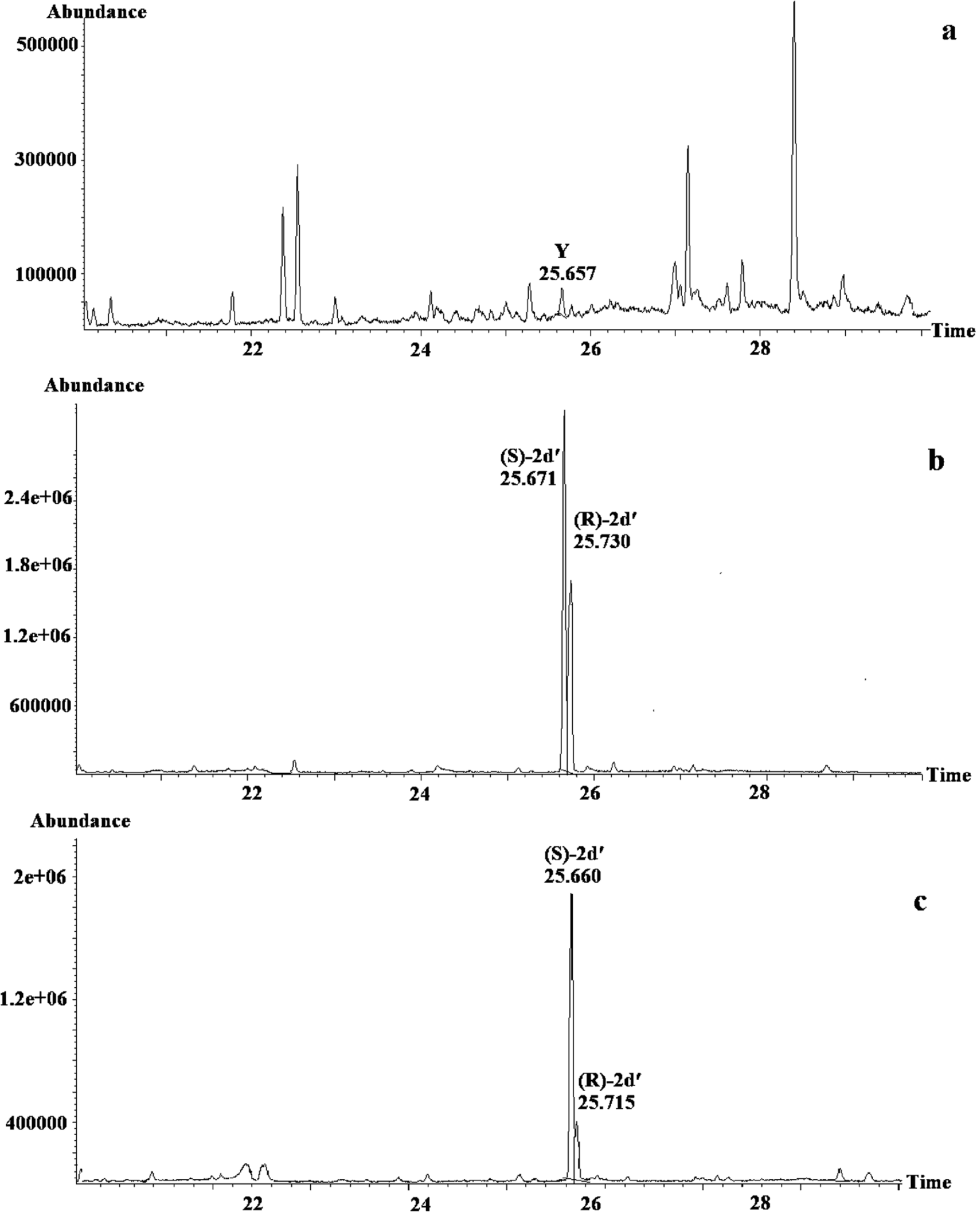
Part of the gas chromatogram (Cyclosil-B) of (**a**) *Whittleia retiella* female extract, (**b**) mixture of (1*S*)-1-methylpropyl (5*Z*)-dec-5-enoate and (1*R*)-1-methylpropyl (5*Z*)-dec-5-enoate, (**c**) (1*S*)-1-methylpropyl (5*Z*)-dec-5-enoate and (1*R*)-1-methylpropyl (5*Z*)-dec-5-enoate mixed with *W. retiella* female extract

The retention time of Y was compared with retention times of a reference ester mixture of known enantiomeric composition as proven by enantioselective GC (Fig. 6 B). By mixing appropriate volumes of the natural extract and the synthetic mixture of enantiomers in the GC-syringe (co-injection), an increase of the peak representing (1*S*)-1-methylpropyl (5*Z*)-dec-5-enoate is noted (Fig. 6c), which shows Y to be (1*S*)-1-methylpropyl (5*Z*)-dec-5-enoate.

**Fig. 7:**
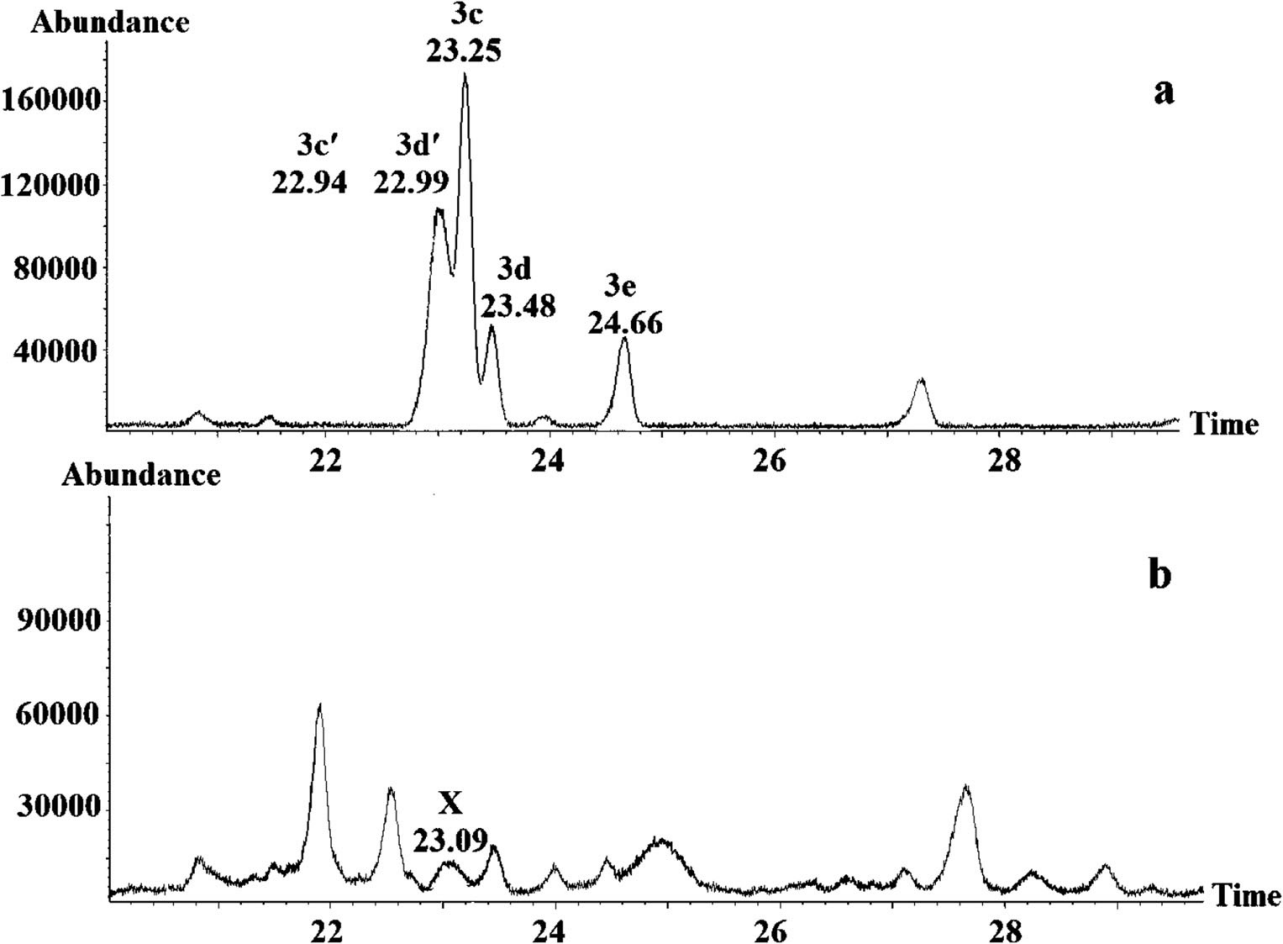
Part of the gas chromatogram (CP-WAX 58 column) of (**a**) 1-methylethyl (4*E*)-dec-4-enoate (3c), 1-methylethyl (4*Z*)-dec-4-enoate (3c’), 1-methylethyl (5*E*)-dec-5-enoate (3d), 1-methylethyl (5*Z*)-dec-5-enoate (3d’), 1-methylethyl dec-6-enoate (3e) and (**b**) *Whittleia retiella* female extract with the natural product X eluting at 23.09 min

**Fig. 8:**
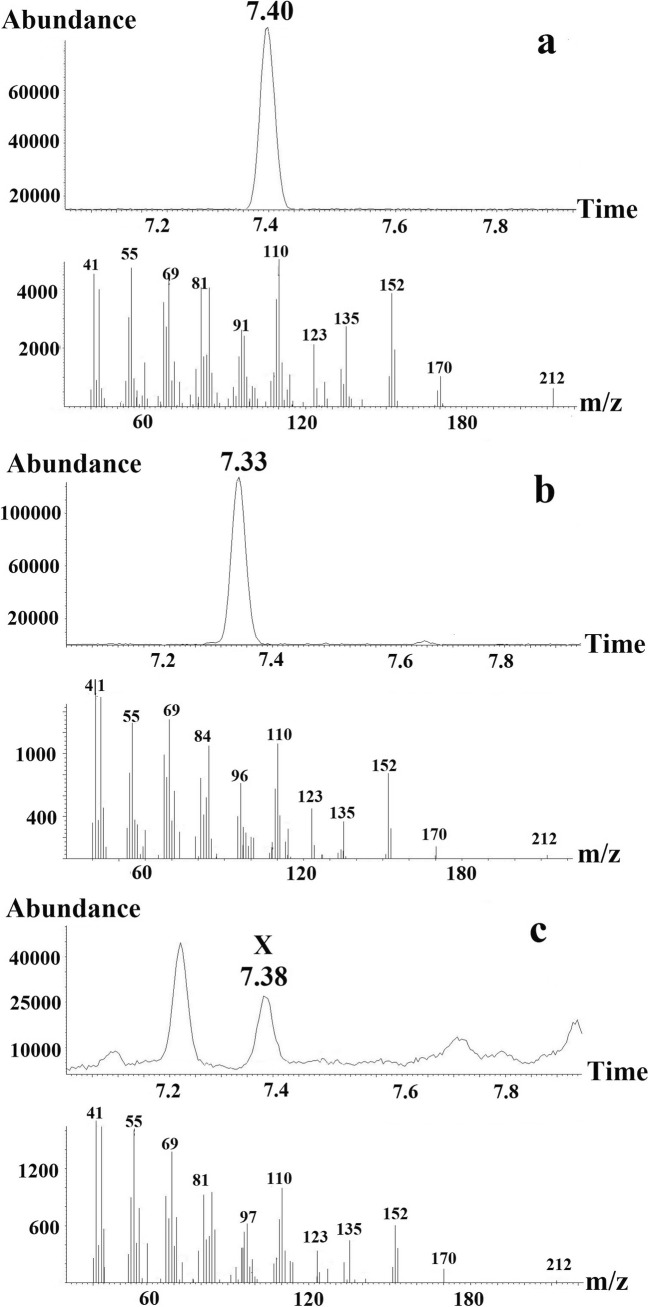
Part of the gas chromatogram (Vf-23 ms column) and the mass spectra of (**a**) 1-methylethyl (5*Z*)-dec-5-enoate, (**b**) 1-methylethyl (4*Z*)-dec-4-enoate, and (**c**) the natural product X, eluting at 7.38 min in the *Whittleia retiella* female extract

For structure elucidation of X, similar GC-MS investigations as for Y were performed using corresponding 1-methylethyl decenoates as reference compounds (Fig. 7). It turned out that the mass spectra of 1-methylethyl (4*Z*)-dec-4-enoate (3c’) and 1-methylethyl (5*Z*)-dec-5-enoate (3d’) were almost identical, and the retention time of both compounds nearly coincided with that of X, but the the latter eluted slightly closer (Fig. 8). However, the other analysed structures could be excluded from the investigation, due to differences in retention times. Consequently, 1-methylethyl (5*Z*)-dec-5-enoate was regarded to be the electrophysiologically active component X, eluting at 7.38 min.

### Field Bioassays

In 2017, three tests at Mulbjerge/Dokkedal revealed attraction of several males to the baits (1: May 15, four males; 2: May 17, one male, and 3: May 22, six males). In 2018, one test at Mulbjerge/Dokkedal (4: May 14) brought two males approaching the baits of series 1, although both series were used, and one test at Kongenshus Hede (5: May 15) brought ten males using only the bait series 2. Males were clearly attracted to the baits and preferentially visited a subset of the baits on display (Table 2 and 3). No approaches were observed to control baits with *n*-hexane. Visits occurred to all baits loaded with the main component (1*S*)-1-methylpropyl (5*Z*)-dec-5-enoate at 100 μg, either as a single component or with additions of 1-methylethyl (5Z)-dec-5-enoate at doses from 10 to 50 μg. At two of the observation periods, visiting males also approached a bait containing 100 μg racemic 1-methylpropyl (5*Z*)-dec-5-enoate and 30 μg 1-methylethyl (5Z)-dec-5-enoate. No attraction was observed to baits containing (1*R*)-1-methylpropyl (5*Z*)-dec-5-enoate alone or in combination with 1-methylethyl (5Z)-dec-5-enoate. No flying males were ever observed apart from those attracted to the baits on display, and no other moth species were observed approaching the baits. Although bioassays often lasted for the better part of a day, all observations of males attracted to baits occurred between 12.00 and 14.00.

**Table 2:** Attraction of *Whittleia retiella* males to bait series 1 at four different field observations in 2017 (Obs 1–3) and 2018 (Obs 4)

Compound	Bait composition											
Isoprop-C5DA			10	30		30	10	30	100		10	30
(rac)-2b-C5DA					200	200	100	100				
*(S)*-2b-C5DA										100	100	100
*(R)*-2b-C5DA		100	100	100								
Blank	BL											
Obs 1 *N* = 4								●		●		●
Obs 2 *N* = 1								●		●		●
Obs 3 *N* = 6								●		●		●
Obs 4 *N* = 2								●				●

Isoprop-C5DA = 1-methylethyl (5*Z*)-dec-5-enoate, 2b-C5DA = 1-methylpropyl (5*Z*)-dec-5-enoate. Different enaniomeric composition of 2b-C5DA (*S*, *R* or racemic) is indicated. Numbers represent μg loading of different synthetic compounds on the baits. Blank baits were loaded with *n-*hexane (hx) only. Black dots represent baits visited by one or more males at each observation, respectively. N = number of approaching males at each observation. At all occasions, the baits were displayed in parallel with bait series 2 (see Table 3)

**Table 3:** Attraction of *Whittleia retiella* males to bait series 2 at four different field observations in 2017 (Obs 1–3) and 2018 (Obs 4–5)

Compound	Bait composition					
Isoprop-C5DA		100		10	30	50
*(S)*-2b-C5DA			100	100	100	100
Blank	BL					
Obs 1 N = 4			●			●
Obs 2 N = 1			●			●
Obs 3 N = 6			●			●
Obs 4 N = 2*						
Obs 5 *N* = 10			●	●	●	●

Isoprop-C5DA = 1-methylethyl (5*Z*)-dec-5-enoate and (*S*)-2b-C5DA = 1*S*-methylpropyl (5*Z*)-dec-5-enoate. Numbers represent μg loading of different synthetic compounds on the baits. Blank baits were loaded with *n*-hexane (hx) only. Black dots represent baits visited by one or more males at each observation, respectively. N = number of approaching males at each observation. At observations 1–4, the baits were displayed in parallel with bait series 1 (see Table 2)

*No visits from males to bait series 2 at this occasion, but to bait series 1, which was displayed in parallel

## Discussion

The baits of each observer were only brought out of the freezers during less than 14 partial days in total over the course of four years, which is less time in the field than many regular field seasons, when baits are normally left continuously in traps. The compounds are stable and have similar molecular weights and likely similar vapour pressures, so we do not expect their relative ratios in the baits to change much over time. The behavioural attraction to the main component was evident, with little possibility to differentiate with regard to the importance of the secondary component in the current setup, and the degree of confidence in the ratios was likely relevant for the situation at hand. Our electrophysiological and behavioural data suggest that the female-produced sex pheromone of the halobiotic psychid *W. retiella* consists of (1*S*)-1-methylpropyl (5*Z*)-dec-5-enoate, and possibly also 1-methylethyl (5Z)-dec-5-enoate. The field bioassays revealed a preference of males towards baits containing (1*S*)-1-methylpropyl (5*Z*)-dec-5-enoate (Table 2 and 3). No visits were recorded to blank baits or to baits with 1-methylethyl (5Z)-dec-5-enoate or the non-natural enantiomer, (1*R*)-1-methylpropyl (5*Z*)-dec-5-enoate, as the main constituents (Table 2). The role of 1-methylethyl (5Z)-dec-5-enoate as a pheromone component could not be conclusively established from these few observations. Baits with only (1*S*)-1-methylpropyl (5*Z*)-dec-5-enoate were among the most preferred baits at two occasions with the highest numbers of visiting males, suggesting that 1-methylethyl (5Z)-dec-5-enoate is not behaviourally active, but there is a possibility that the presence of the latter compound among adjacent baits may have contributed to the attraction. There is a recorded case where male moths, once activated by the complete blend of a female pheromone, display lower selectivity during the subsequent search for the pheromone source (Karpáti et al. 2013).

The attraction to the baits containing the *S*-enantiomer was evident, as opposed to the lack of approaches to controls or baits lacking the *S*-enantiomer, whereas the relative numbers of approaches to different ratios of the secondary component were not possible to distinguish quantitatively for individual males, preventing any form of rigorous statistical testing.

Pheromone identifications in bagworms are still scarce, and *W. retiella* is the fifth species for which a detailed investigation has been performed. All psychid species analysed so far use esters of long-chain fatty acids as sex pheromones. Leonhardt et al. (1983) identified (*R*)-1-methylbutyl decanoate as sex pheromone of *Thyridopteryx ephemeraeformis* (Haworth), and the same compound and four additional esters were found to constitute the sex pheromone of *Oiketicus kirbyi* (Guilding) (Rhainds et al. 1994). Subchev et al. (2000) reported 1-methylethyl octanoate as sex pheromone of *Megalophanes viciella* (Denis & Schiffermüller). More recently, Gries et al. (2006) revealed two antennally active compounds in females of *Clania variegata* (Snellen), and the most abundant compound was identified as (1*S*)-1-ethyl-2-methylpropyl 3,13-dimethylpentadecanoate. The use of long-chain fatty acid esters as sex pheromone components outside the bagworm family seems to be restricted, and so far only revealed for species belonging to the subfamily Procridinae within the Zyganidae, *e.g.* (1*S*)-1-methylpropyl (7*Z*)-tetradecenoate in *Theresimima ampellophaga* Bayle-Barelle (Subchev et al. (1998), and (1*R*)-1-methylpropyl (7*Z*)-dodecenoate and (1*R*)-1-methylpropyl (9*Z*)-tetradecenoate in *Illiberis rotundata* Jordan (Subchev et al. 2009). In both Psychidae and Procridinae enantiomeric specificity in the pheromone channel is apparent, whereas the opposite enantiomers of a pheromone component are inactive or even inhibit male attraction.

Monitoring with an efficient sex pheromone is a valuable conservation tool when gathering information about the geographic distribution and habitat requirements of rare and threatnend insect species. Detection of *W. retiella* populations in salt marsh habitats would be greatly facilitated by implementing a monitoring system using baits loaded with (2*S*)-1-methylpropyl (5*Z*)-dec-5-enoate. The species has been recorded in Great Britain, France, Germany, Belgium, Holland, Denmark, and Sweden (Rickert et al. 2009), but high resolution data on its distribution are lacking. Because *W. retiella* is small and only flies at sunny days during a short period of spring, it may have been overlooked. This identification of the pheromone of *W. retiella* included few females, and the pheromone is produced in minute amounts. Thus, further optimisiation of the pheromone may be needed, *e.g.* testing different doses of (2*S*)-1-methylpropyl (5*Z*)-dec-5-enoate applied on septa and the role of 1-methylethyl (5Z)-dec-5-enoate as potential pheromone component to improve the attraction of males to baits in the field.

## Electronic Supplementary Material

ESM 1 (DOCX 24 kb)
