# Supplementary material for: Identification and Synthesis of Putative Pheromone Components of the Threatened Salt Marsh Bagworm Moth, Whittleia retiella (Lepidoptera: Psychidae)
Source: J Chem Ecol. 2020 Feb 13;46(2):115–27. doi: 10.1007/s10886-020-01145-x (PMC7056680; doi:10.1007/s10886-020-01145-x)
Supplement: Supplementary file 1 — (DOCX 24 kb) [file 10886_2020_1145_MOESM1_ESM.docx]

IDENTIFICATION AND SYNTHESIS OF PUTATIVE PHEROMONE COMPONENTS OF A THREATENED SALT MARSH BAGWORM, *WHITTLEIA RETIELLA* (LEPIDOPTERA: PSYCHIDAE)

RIZAN RAHMANI^a^ DAVID CARRASCO^b,c^, GLENN P. SVENSSON^d^, HARTMUT ROWECK^e^, NILS RYRHOLM^f^, MATTIAS C. LARSSON^b^* AND ERIK HEDENSTRÖM ^a^*

^a^Eco-Chemistry, Department of Chemical Engineering, Mid Sweden University, SE-851 70 Sundsvall, Sweden

^b^ Dept. of Plant Protection Biology, Swedish University of Agricultural Sciences, Box 102 SE-230 53 Alnarp, Sweden

^c^Present address: MIVEGEC, IRD, CNRS, University of Montpellier, Montpellier, France

^d^Department of Biology, Lund University, Lund, Sweden

^e^Christian Albrecht University, Faculty of Agricultural and Nutritional Sciences, Olshausenstraße 75, R. 220, Kiel, Germany

^f^ Dept. of Electronics, Mathematics and Natural Sciences, Faculty of Engineering and Sustainable Development, University of Gävle, S-801 76 Gävle, Sweden

*Corresponding author email address: [erik.hedenstrom@miun.se](mailto:erik.hedenstrom@miun.se) and [mattias.larsson@slu.se](mailto:mattias.larsson@slu.se)

SUPPLEMENTARY SECTION

**Chemical Analysis.**

All synthetic compounds were analysed on a GC-FTIR instrument equipped with a Varian gas chromatograph and a 30-m x 0.25-mm-ID fused silica capillary column with a HP-5ms phase, coupled to a Nicolet Fourier Transform Infra-Red instrument. Gas chromatography was run with a temperature program at an initial temperature of 50 °C·, then programmed to 230 °C at 10 °C·min^−1^.

NMR spectra were recorded on a Bruker Avance 500 (500 MHZ ^1^H, 125.8 MHZ ^13^C) spectrometer using CDCl_3_ as the solvent; all shifts are reported in ppm. One-dimensional ^1^HNMR and ^13^CNMR in CDCl_3_ at 25°C were acquired for all synthetic compounds.

Optical rotations were measured on a Perkin–Elmer 341 Polarimeter in an 1 dm cell at 546 nm.

DILASMS was applied by direct injection on an Agilent 6520 Accurate-Mass Q-TOF quadrupole-time of flight mass spectrometer in ESI and positive mode.

**1-Methylpropyl** **(4*E*)-dec-4-enoate. (**4*E*)-Dec-4-enoic acid (19 mg, 0.11mmol) was dissolved in 3 mL racemic butan-2-ol, one drop of H_2_SO_4_ (2M) was added at room temperature, and the mixture was stirred overnigh. The reaction was quenched after 24 h, and the solution was extracted with 3×15 mL Et_2_O, washed with 10 mL of brine and dried with Mg_2_SO_4_ (Fujii and Yoshifuji 1978)**.** The product was obtained in 35.9% yield as a liquid. DILASMS [M + Na]^+^ = 249.1825; IR 2937-1747-1367-1241-1169-974 ^1^HNMR (CDCl_3_) δ 0.88 (3H, t, *J* = 6.8), 0.89 (3H, t, *J* =7), 1.92 (3H, d, *J* = 6), 1.23-1.35 (6H, m), 1.48-1.63 (2H, m), 1.94-1.99 (2H, m), 2.27-2.37 (2H, m), 2.39-2.43(2H, m), 4.84 (1H, sextet, *J* = 6.3), 5.36-5.51 (2H, m); ^13^CNMR mixture of E and Z; (CDCl_3_) δ 9.69, 14.04, 19.50, 22.51, 27.61, 28.05, 28.80, 29.07, 29.11, 30.30, 31.32, 31.34, 32.45, 32.47, 34.08, 34.75, 72.03, 127.50, 127.93, 131.76, 132.16, 173.03; MS (EI) m/z (relative intensity): 226 (M+, 3.6%), 170 (29), 152 (64), 135 (54), 123 (35), 110 (89), 96 (56), 84 (89), 69 (100), 55 (82), 41 (96).

**1-Methylpropyl** **(4*Z*)-dec-4-enoate.** (4*Z*)-Dec-4-en-1-ol **(**33 mg, 0.21mmol) was dissolved in 5 mL acetone at 0 °C, and Jones reagent was added dropwise until the color changed to orange. Subsequently, a spoon of celite and a few drops of propan-2-ol were added. The reaction was quenched, and the mixture was extracted with 3×10 mL Et_2_O, washed with 10 mL of brine, and dried with Mg_2_SO_4_ (Bowden et al. 1946). To (4*Z*)-dec-4-enoic acid (23.5 mg, 0.14 mmol) dissolved in 5 mL racemic butan-2-ol, one drop of H_2_SO_4_ (2M) was added at room temperature, and the solution was stirred overnight. The reaction was quenched after 24 h, and the mixture was extracted with 3×15 mL Et_2_O, washed with 10 mL of brine, and dried with Mg_2_SO_4_ (Fujii and Yoshifuji 1978). The target product was isolated in 23.8% yield as a liquid. DILASMS [M + Na]^+^ = 249.1825; IR 2941, 1747, 1458, 1366, 1238, 1168; ^1^HNMR (CDCl_3_) δ 0.89 (3H, t, *J* = 6.8), 0.89 (3H, t, *J* =7.5), 1.2 (3H, d, *J* =6), 1.23-1.38 (6H, m), 1.48-1.64 (2H, m), 2.02-2.06 (2H, m), 2.26-2.37 (4H, m), 4.85(1H, sextet, *J* = 6.3), 5.30-5.44 (2H, m); ^13^CNMR (CDCl3) δ 9.69, 14.04, 19.49, 22.56, 22.94, 27.17, 28.82, 29.30, 31.49, 34.77, 120.08, 127.44, 131.45, 172.97; MS (EI) m/z (relative intensity): 170 (3), 152 (27), 135 (63), 123 (53), 110 (34), 96 (87), 84 (55), 69 (88), 55 (100), 41 (85).

**1-Methylpropyl** **(5*E*)-dec-5-enoate.** The title compound was synthesised as above for 1-methylpropyl (4*Z*)-dec-4-enoate but from (5*E*)-dec-5-en-1-ol and racemic butan-2-ol DILASMS [M + Na]^+^ = 249.1825; IR 2943, 1746, 1169, 973; ^1^HNMR (CDCl_3_) δ 0,89 (6H, m), 1,2 (3H, d, *J* = 6), 1,28-1,35 (4H, m), 1,48-1,71 (4H, m), 1,96-2,04 (4H, m), 2,27 (2H, t, *J* = 7.8), 4,84 (1H, sextet, *J* = 6), 5,33-5,45 (2H, m); ^13^CNMR (CDCl_3_) δ 9.7, 13.94, 19.51, 22.18, 24.95, 28.83, 31.73, 31.95, 32.24, 34.03, 71.91, 128.96, 131.58, 173.50; MS (EI) m/z (relative intensity): 226 (M+, 6%), 170 (38), 153 (79), 135 (73), 123 (39), 110 (100), 96 (52), 84 (87), 69 (76), 55 (90), 41 (86).

**(1*S*)-1-Methylpropyl** **(5Z)-dec-5-enoate.** (5*Z*)-Dec-5-en-1-ol (59 mg, 0. 39 mmol) was dissolved in 5 mL acetone at 0 °C. Jones reagent was added dropwise until the color changed to orange. A spoon of celite and a few drops of propan-2-ol were added. The reaction was quenched, and the mixture was extracted with 3×10 mL Et_2_O. The extract was washed with 10 mL of brine and dried over Mg_2_SO_4_ (Bowden et al. 1946)**.** The obtained (5*Z*)-dec-5-enoic acid was used in a lipase catalysed esterification as follows: 50 mg crude lipase CRL (stored in the fridge) was added to 3 mL of *n*-hexane containing molecular sieves (3 Å), 0.12 mM (2*S*)-butan-2-ol, and 0.059 mM (5*Z*)-dec-5-enoic acid. The mixture was stirred at room temperature for 9 days when the enzyme was removed by filtration, and the remaining unreacted acid was separated. The produced ester were purified by flash chromatography, yielding 43% of the target product as a liquid. DILASMS [M + Na]^+^ =249.1825; IR 2949, 1746, 1651, 1542, 1460, 1174, 1101, 1008, 759; ^1^HNMR (CDCl_3_) δ 0.89 (6H, t, *J* = 7.5), 1.2 (3H, d, J = 6.5), 1.29-1.33 (4H, m), 1.5-1.71 (4H, m), 1.2-2.1 (4H, m), 2.29 (2H, t, *J* = 7.8), 4,85 (1H, sextet, *J* = 6.2), 5.30-5.43(2H, m); ^13^CNMR (CDCl_3_) δ 9.70, 13.97, 19.49, 22.34, 25.08, 26.58, 26.92, 28.82, 31.89, 34.15, 71.95, 128.74, 131.02, 173.41; MS (EI) m/z (relative intensity): 226 (M+, 5%), 170 (39), 152 (73), 135 (76), 123 (41), 110 (99), 96 (52), 84 (89), 69 (85), 55 (100), 41 (97); [α] _D_^20^ = +12.1 (c = 0.82, CHCl_3_).

**(1*R*)-1-Methylpropyl (5*Z*)-dec-5-enoate.** The title compound was synthesised as above for (1*S*)-1-methylpropyl (5Z)-dec-5-enoate but from (5*Z*)-dec-5-en-1-ol and (2*R*)-butan-2-ol. DILASMS, IR, ^1^HNMR, ^13^CNMR, and MS data were identical with the data for (1*S*)-1-methylpropyl (5Z)-dec-5-enoate. [α]_D_^20^ = –13.8 (c = 0.82, CHCl_3_).

***rac*-1-Methylpropyl** **(5Z)-dec-5-enoate.** The title compound was synthesised as above for 1-methylpropyl (4*Z*)-dec-4-enoate but from (5*Z*)-dec-5-en-1-ol and racemic butan-2-ol. DILASMS, IR, ^1^HNMR, ^13^CNMR and MS data were identical with the data for (1*S*)-1-methylpropyl (5Z)-dec-5-enoate.

# 1-Methylpropyl (6*E/Z*)-dec-6-enoate. 6-Decenoic acid was synthesised from 1 g (2.18 mmol) (5-carboxypentyl) triphenylphosphonium bromide (Wittig reagent), 4.37 mL ( 4.37 mmol) sodium hexamethyldisilylamide (NaHDMS) and 196.5 μl (2.18 mmol) butanal in 25 ml THF according to the literature (Wube et al. 2011). To 276 mg (1.62 mmol) 6-decenoic acid, dissolved in 15 mL racemic butan-2-ol, one drop of H_2_SO_4_ (2M) was added at room temperature, and the mixture was stirred overnight. The reaction was quenched after 24 h, and the solution was extracted with 3×15 ml Et_2_O, washed with 10 mL of brine, and dried with Mg_2_SO_4_ (Fujii and Yoshifuji 1978)_._ The ester was obtained in 36.3% yield as a liquid. DILASMS [M + Na]^+^ = 249.1822; IR 2929, 2863, 1731, 1709, 1457, 1377, 1176, 1115, 1096, 968, 721, 695; HNMR (CDCl_3_) δ 0.87-0.91 (6H, m), 1.19 (3H, d, *J* = 6), 1.33-1.44 (4H, m), 1.49-1.69 (4H, m), 1.93-2.09 (4H, m), 2.28 (2H, t, *J* = 7.5), 4.48 (1H, sextet, *J* = 6.5), 5.32-5.41 (2H, m); ^13^CNMR (CDCl_3_) δ 9.85, 13.94, 19.65, 22.99, 24.88, 26.99, 28.96, 29.24, 29.43, 34.78, 72.08, 129.49, 130.29, 173.63; MS (EI) m/z (relative intensity): 226 (M+, 4%), 170 (27), 153 (100), 135 (21), 123 (37), 110 (61), 96 (37), 84 (82), 69 (70), 55 (99), 41 (92).

**1-Methylethyl** **(4*E*)-dec-4-enoate.** The title compound was synthesised as above for 1-methylpropyl (4*E*)-dec-4-enoate but from (4*E*)-dec-4-enoic acid and propan-2-ol. DILASMS [M + Na]^+^ = 235.167; IR 2936, 1748, 1369, 1241, 1166, 1119, 967; ^1^HNMR (CDCl_3_) δ 0.88 (3H, t, *J* = 7.0), 0.89 (6H, m), 1.22 (3H, d, *J* = 6.5), 1.21-1.34 (6H, m), 1.94-1.98 (2H, q, *J* = 5), 2.28-2.32 (4H, m), 5.00 (1H, septet, *J* = 6.2), 5.36-5.43 (2H, m); ^13^CNMR (CDCl_3_) δ 14.05, 21.86(2C), 22.52, 28.02, 29.13, 31.34, 32.47, 34.75, 67.43, 127.93, 131.77, 172.82; MS (EI) m/z (relative intensity): 212 (M+, 12%), 170 (20), 152 (82), 135 (46), 123 (41), 110 (95), 96 (61), 84 (89), 69 (100), 41 (90).

**1-Methylethyl (4*Z*)-dec-4-enoate.** (Zhang et al. 2015). The title compound was synthesised as above for 1-methylpropyl (4*Z*)-dec-4-enoate but from (4*Z*)-decenol and propan-2-ol. MS (EI) m/z (relative intensity): 212 (M+, 9%), 170 (20), 152 (82), 135 (44), 123 (41), 110 (94), 96 (59), 84 (86), 69 (100), 41 (90).

**1-Methylethyl** **(5*E*)-dec-5-enoate.** The title compound was synthesised as above for 1-methylpropyl (4*Z*)-dec-4-enoate but from (5*E*)-dec-5-en-1-ol and propan-2-ol. DILASMS [M + Na]^+^ = 235.1668; IR 2937, 1747, 1371, 1234, 1120, 966; ^1^HNMR (CDCl_3_) δ 0.88 (3H, t, *J* = 7.0), 1.23 (6H, d, *J* = 6.5), 1.28-1.33 (4H, m), 1.64-1.72 (2H, m), 1.96-2.03 (4H, m), 2.26 (2H, t, *J* = 7.8), 5.0 (1H, septet, *J* = 6.25), 5.33-5.45 (2H, m); ^13^CNMR (CDCl_3_) δ 13.94, 21.86(2C), 22.19, 24.89, 31.73, 31.93, 32.24, 34.03, 67.34, 128.93, 131.58, 173.32; MS (EI) m/z (relative intensity): 212 (M+, 15%), 170 (22), 152 (69), 135 (55), 123 (40), 110 (100), 96 (52), 81 (84), 69 (86), 55 (97), 41 (88).

**1-Methylethyl** **(5*Z*)-dec-5-enoate.** The title compound was synthesised as above for 1-methylpropyl (4*Z*)-dec-4-enoate but from (5Z)-dec-5-en-1-ol and propan-2-ol. DILASMS [M + Na]^+^ = 235.1667; IR 2948, 1747, 1459, 1373, 1230, 1120; ^1^HNMR (CDCl_3_) δ 0.88-0.91 (3H,m), 1.27 (6H, s), 1.29-1.33 (4H,m), 1.67 (2H, quintet, *J* = 7.5), 1.99-2.09 (4H, m), 2.27 (2H, t, *J* = 7.5), 5.0 (1H, septet, *J* = 6.25), 5.3-5.43 (2H,m); ^13^CNMR (CDCl_3_) δ 13.98, 21.86 (2C), 22.35, 25.02, 26.55, 26.93, 31.90, 34.14, 67.39, 128.48, 131.03, 173.25; MS (EI) m/z (relative intensity): 212 (M+, 11%), 170 (21), 152 (75), 135 (53), 123 (41), 110 (99), 96 (54), 81 (84), 69 (89), 55 (100), 41 (97).

**1-Methylethyl** **(6*E/Z*)-dec-6-enoate.** The title compound was synthesised as above for 1-methylpropyl (6*E/Z*)-dec-6-enoate but from 6-decenoic acid and propan-2-ol. DILASMS [M + Na]^+^ = 235.1668; IR 2948, 1747, 1373, 1120; ^1^HNMR (CDCl_3_) δ 0.9 (3H, t, *J* = 7.3), 1.23 (6H, d, *J* =6), 1.33-1.41(4H, m), 1.58-1.68 (2H, m), 1.93-2.07 (4H, m), 2.27 (2H, t, *J* = 7.5), 5.0 (1H, m), 5.33-5.4 (2H, m); ^13^CNMR (CDCl_3_) δ 13.95, 22.85 (2C), 23.00, 24.83, 27.00, 29.34, 29.44, 34.77, 67.51, 129.52, 130.30, 173.47; MS (EI) m/z (relative intensity): 212 (M+, 8%), 170 (14), 153 (56), 133 (18), 123 (38), 110 (61), 96 (35), 84 (75), 69 (72), 55 (100), 41 (87).

REFERENCES

Bowden K, Heilbron IM, Jones ERH, Weedon BCL (1946) Researches on acetylenic compounds. Part I. The preparation of acetylenic ketones by oxidation of acetylenic carbinols and glycols. J Chem Soc (0):39-45. DOI: 10.1039/jr9460000039.

Fujii T, Yoshifuji S (1978) Lactams. XIV. cis-trans Isomerization in the 5-ethyl-2-oxo-4-piperidine-acetic acid system under Fischer-Speier esterification conditions. Chem Pharm Bull 26:2253-2257. DOI: 10.1248/cpb.26.2253.

Wube AA, Huefner A, Thomaschitz C, Blunder M, Kollroser M, Bauer R, Bucar F (2011) Design, synthesis and antimycobacterial activities of 1-methyl-2-alkenyl-4(1H)-quinolones. Bioorg Med Chem 19:567-579. DOI: 10.1016/j.bmc.2010.10.060.

Zhang C, Santiago CB, Kou L, Sigman MS (2015) Alkenyl carbonyl derivatives in enantioselective redox relay Heck reactions: Accessing α,β-unsaturated systems. J Am Chem Soc 137: 7290-7293. DOI:10.1021/jacs.5b04289.
